# Mutational profiles of different macroscopic subtypes of colorectal adenoma reveal distinct pathogenetic roles for KRAS, BRAF and PIK3CA

**DOI:** 10.1186/s12876-014-0221-y

**Published:** 2014-12-31

**Authors:** Li-Chun Chang, Han-Mo Chiu, Chia-Tung Shun, Jin-Tung Liang, Jaw-Town Lin, Chien-Chuan Chen, Yi-Chia Lee, Ming-Shiang Wu

**Affiliations:** Department of Internal Medicine, National Taiwan University Hospital, Bei-Hu branch, Taipei, Taiwan; Graduate Institute of Clinical Medicine, College of Medicine, National Taiwan University, Taipei, Taiwan; Department of Internal Medicine, National Taiwan University Hospital, Taipei, Taiwan; Department of Pathology, National Taiwan University Hospital, Taipei, Taiwan; Department of Surgery, National Taiwan University Hospital, Taipei, Taiwan

**Keywords:** Gene mutation, Non-polypoid colorectal neoplasm, EGFR

## Abstract

**Background:**

Investigations of genetic alterations and correlations with histology or morphology could provide further insights into colorectal carcinogenesis. Nevertheless, such genetic changes were less investigated in adenoma stage and a comprehensive survey of oncogenic mutations in EGFR signaling pathway according to different morphologic subtypes has not been performed.

**Methods:**

A total of 94 neoplasms, including 34 polypoid adenoma, 16 lateral spreading tumors-granular (LST-G), 20 non-granular LST (LST-NG), and 24 depressed tumors, were subjected for mutational analysis of KRAS (exon 2), BRAF (exon 11 and 15), PIK3CA (exon 9 and 20), AKT (exon 4), EGFR (exon 18–24) and HER2 (exon18-24).

**Results:**

KRAS mutation was noted more frequently in LST (13/36, 36.1%) than polypoid neoplasms (5/34, 14.7%, p = 0.041). When comparing with LST-NG, LST-G had a significantly higher frequency of KRAS mutation. (9/16, 56.3% *vs.* 4/20, 20.0%, p = 0.024). BRAF mutation (V600E) was found in 2 of 36 (5.6%)LSTs and 1 of 34 (2.9%) polypoid lesions. The two LST lesions with BRAF mutation were pathologically proven to be serrated adenoma. PIK3CA mutation (exon 9 E545K) was identified only in LST (5/36, 13.9%). Mutations in KRAS, BRAF or PIK3CA occurred in a mutually exclusive manner. All mutations were absent in the specimens obtained from depressed type neoplasms.

**Conclusions:**

Three different macroscopic subtypes of colorectal neoplasms display distinct carcinogenetic pathways in EGFR networking. Further molecular studies of CRCs should take macroscopic subtypes into consideration and highlight the importance of consensus and communication between endoscopic and pathologic diagnosis.

## Background

Colorectal cancer (CRC) is the most frequent malignancy of gastrointestinal tract. With respect to pathogenesis and therapeutic responses, CRC is regarded as a heterogenous disease. Macroscopically, CRC can be classified into polypoid and nonpolypoid subtypes [1]. The latter usually presents as flat or depressed tumors and is further subcategorized into depressed type, flat tumor less than 1.0 cm (0-IIa) and laterally spreading tumor (LST). Among nonpolypoid variants, depressed subtypes have the most aggressive behavior and represent the main lesions of de novo pathway [2-7]. Such variants are more difficult to identify by endoscopy and initially reported only in Japan. However, with the advance of image enhanced endoscopic techniques, the incidence of non-polypoid neoplasms has been rising and now accounts for 20-30% of CRC [7,8]. The unique clinicopathologic characteristics and biological behaviors for different macroscopic morphology suggest that distinct carcinogenetic pathways might exist for various subtypes of CRC.

The classic adenoma-carcinoma sequence of CRC was defined on histologic grounds and had been the cornerstone of current screening, surveillance and prevention. Polypoid adenoma is the main precursor lesion in such classical pathways. Serial genetic changes in APC, KRAS, and p53 genes had been reported in this sequence [9]. Investigations of genetic alterations and correlations with histology or morphology could provide further insights into colorectal carcinogenesis. A recent example is the identification of oncogenic mutation of BRAF in serrated pathway [10]. Furthermore, a better understanding of the molecular mechanisms underlying the development of different CRCs would open the way for patient-specific therapy. This is reflected in the current practice that anti-epidermal growth factor receptor antibody therapy is no longer offered to CRC patients with mutant KRAS [11]. Moreover, nevertheless the fecal DNA testing was developed to detect significant neoplastic lesions, the precise genetic change of nonpolypoid neoplasm remains elusive and therefore such neoplasms may be missed by these tests [12]. Collectively, elucidation of genetic mechanisms by which these alterations affect colorectal carcinogenesis might have a profound impact on more effective strategies for screening, diagnosis and treatment of CRCs [13,14].

Epidermal growth factor receptor (EGFR) signaling influences multiple downstream pathways, including Ras/Raf/MAPK and phosphatidylinositol 3’-kinase (PI3K)/AKT pathways. Alterations of signaling in this pathway had been reported to affect cell proliferation, survival and apoptosis of a variety of malignancies [14]. A substantial portion of CRC was observed to harbor nonoverlapping somatic mutations of KRAS, BRAF, PIK3CA and AKT genes [15-23]. Nevertheless, such genetic changes were less investigated in adenoma stage and a comprehensive survey of oncogenic mutations in EGFR signaling pathway according to different morphologic subtypes has not been performed. In this study, we aimed to assess the frequency and distribution of EGFR pathway alterations in three subtypes of colorectal neoplasms. Some subcategories of 0-IIa lesions, the so called laterally spreading tumor (LST), extend laterally and circumferentially rather than vertically along the colonic wall and the frequency of invasive carcinoma is known to be less than that of polypoid lesions with similar size [14,15]. Though it is classified as “non-polypoid” neoplasm as a whole with depressed lesions recently, its clinicopathological characteristics are distinct from depressed (0-IIc) or flat and depressed (0-IIa + IIc or 0-IIc + IIa) lesions [6,7]. LSTs are considered as less invasive as they rarely become invasive till the size of 3 cm or more [6,15]. Among LSTs, granular type (LST-G) and flat type (LST-NG) are also different. Malignant transformation is more common in LST-NG at smaller size with higher risk of multi-focal submucoal invasion in comparison with LST-G [15]. Some different genetic alterations were also observed between polypoid, LST-G and LST-NG [16,17]. Therefore, it is reasonable to speculate a unique biological behavior or molecular basis of malignant transformation in such non-polypoid neoplasms.

Among different phenotypes of LST, granular type and flat type were reported to have different frequency of KRAS mutation and other genetic or epigenetic changes [16,17]. However, concurrent analysis of KRAS and BRAF mutation for such lesions has never been conducted before. In this study, we aim to elucidate the frequency of KRAS mutation and BRAF mutation in polypoid, flat and depressed colorectal neoplasms and compare these morphological counterparts each other.

## Methods

### Patient samples

**Figure 1:**
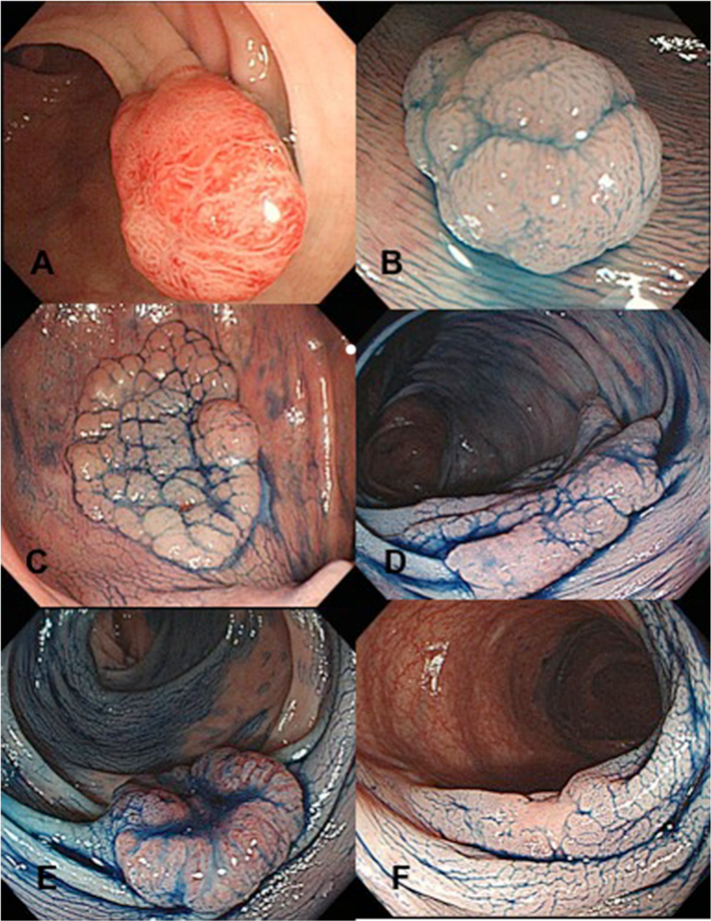
**Macroscopic classification of colorectal neoplasm. A**: 0-Ip, **B**: 0-Is, **C**: LST-G, **D**: LST-NG, **E**: 0-IIa + IIc, **F**: 0-IIc.

The study protocol was approved by institutional review board of National Taiwan University Hospital, and informed consent was obtained from all patients. We prospectively recruited 85 patients who underwent endoscopic or surgical resection of colorectal neoplasms from September 2006 to December 2008. The endoscopic morphology of colorectal tumors was mainly based on Paris and Japanese Research Society classifications with some modifications of subtypes as we have described elsewhere [1]. Lesions with 0-Is or 0-Ip were classified into “protruded” subtype (Figure 1, A & B). LSTs were defined as lesions ≥10 mm in diameter with a low vertical axis extending laterally along the interior luminal wall. They were further subdivided into granular (LST-G) or nongranular (LST-NG) subtypes according to macroscopic findings: LST-G has even or uneven nodules on the surface whereas LST-NG type has a smooth surface (Figure 1, C & D). Lesions categorized as 0-IIc, 0-IIc + IIa or 0-IIa + IIc were depressed subtype (Figure 1, E & F). A total of 94 neoplasms were subjected for further analyses. These included 34 polypoid subtype, 36 LSTs (16 LST-G, 20 LST-NG), and 24 depressed subtype.

### Histopathological assessment

Hematoxylin and eosin stained slides prepared from routinely processed paraffin-embedded tissue samples were assessed. The histopathological analysis of each tumor was done according to the WHO criteria [24]. Advanced colorectal neoplasia was defined as those lesions with one of the following criteria: lesions larger than 10 mm in diameter, lesions with a villous component, high-grade dysplastic (HGD) lesions or carcinoma in situ (CIS), and lesions with invasive features. All specimens were reviewed by the same pathologist (Chia-Tung Shun), who was unaware of the colonoscopic findings, medical history and the results of genetic analysis.

### DNA extraction

DNA was extracted from surgical or endoscopic specimens. Briefly, tumor cell-rich areas in 0.1% methylene blue-stained 10-μm sections under microscopy were scratched with 20 gauge needles and deparaffinized. Recovered tissues were incubated in 1X polymerase chain reaction (PCR) buffer containing proteinase K solution. After heat inactivation, the extracted DNAs were subjected for sequencing analyses.

### Mutation analyses

Intron-based PCR primers were used to amplify the targeted exons of KRAS, BRAF, HER2, EGFR, PIK3CA and AKT genes based on previously published sequences. The primers were as follows (forward and reverse, respectively): BRAF exon 11 (5’-TTCTGTTTGGCTTGACTTGACTT-3’ and 5’-ACTTGT CACAATGTCACCTT-3’) and exon 15 (5’-TGCTTGCTCTGATAGGAAAAT G-3’ and 5’-AGCATCTCAGGGCCAAAAAT-3’); [23] KRAS exon 2 (5’-CTGAAAATGACTGAATATAAACTTGT-3’ and 5’-ATATGCATATTAAAACAA GATTTACC-3’); [24] AKT exon 4 (5’-CACACCCAGTTCCTGCCT-3’ and 5’-CCTGGTGGGCAAAGAGGGCT-3’); [25] PIK3CA exon 9 (5’-TCAGCAGTTACTATTCTGTGACTGG-3’ and 5’-GTAAAACGACGGCCAGTTGCTGAGATCAGCCAAATTCA-3’) and exon 20 (5’-GTAAAACGACGGCCAGTGACATTTGAGCAAAGACCTGAAG-3’ and 5’-TGGATTGTGCAATTCCTATGC-3’); [26] EGFR exon 18 (5’-AGCATGGTGAGGGCTGAGGTGAC-3’ and 5’-ATATACAGCTTGCAAGGACTCTGG-3’), exon 19 (5’-CCAGATCACTGGGCAGCATGTGGCACC-3’ and 5’-AGCAGGGTCTAGAGCAGAGCAGCTGCC-3’), exon 20 (5’-GATCGCATTCATGCGTCTTCACC-3’ and 5’-TTGCTATCCCAGGAGCGCAGACC-3’), exon 21 (5’-TCAGAGCCTGGCATGAACATGACCCTG-3’ and 5’-GGTCCCTGGTGTCAGGAAAATGCTGG-3’), exon 22 (5’-AATTAGGTCCAGAGTGAGTTAAC-3’ and 5’-ACTTGCATGTCAGAGGATATAATG-3’), exon 23 (5’-CATCAAGAAACAGTAACCAGTAATG-3’ and 5’-AAGGCCTCAGCTGTTTGGCTAAG-3’), exon 24 (5’-TTGACTGGAAGTGTCGCATCACC-3’ and 5’-CATGTGACAGAACACAGTGACATG-3’); [27] HER2 exon 18 (5’-GTGAAGTCCTCCCAGCCCGC-3’ and 5’-CTCCCATCAGAACTGCCGACC-3’), exon 19 (5’-TGGAGGACAAGTAATGATCTCCTGG-3’ and 5’-AAGAGAGACCAGAGCCCAGACCTG-3’), exon 20 (5’-GCCATGGCTGTGGTTTGTGATGG-3’ and 5’-ATCCTAGCCCCTTGTGGACATAGG-3’), exon 21 (5’-GGACTCTTGCTGGGCATGTGG-3’ and 5’-CCACTCAGAGTTCTCCCATGG-3’), exon 22 (5’-CCATGGGAGAACTCTGAGTGG-3’ and 5’-TCCCTTCACATGAGGTGG-3’), exon 23 (5’-AGACTCCTGAGCAGAACCTCTG-3’ and 5’-AGCCAGCACAGCTCAGCCAC-3’), and exon 24 (5’-ACTGTCTAGACCAGACTGGAGG-3’ and 5’-GAGGGTGCTCTTAGCCACAGG-3’) [28]. PCR was performed in a 20-μl volume containing 100 ng of template DNA, 10 × PCR buffer; 0.25 mM deoxynucleoside triphosphate (dNTP), 20 pmol primers, and 1.5 U Taq DNA polymerase (Takara Shuzo, Kyoto, Japan). PCR products were electrophoresed on 2% agarose gels, purified, and both strands were directly sequenced using the BigDye Terminator v3.1 cycle sequencing kit, followed by analysis with an Applied Biosystems 3700 automated sequencer (Applied Biosystems, Foster City, CA, USA). All suspected mutations were confirmed by independent PCR amplifications and sequenced in both directions.

### Statistical methods

Statistical analysis was done using SAS program (version 9.1, Cary, NC, U.S.A.). Noncontinuous variables were analyzed with chi-square test or Fischer’s exact test as indicated. Continuous variables were analyzed using two-sided *t* tests.

## Results

### Demographic and clinicopathological characteristics of polypoid, LST and depressed neoplasms

A total of 94 colorectal neoplasms were collected from 85 patients, including 34 polypoid lesions, 36 LST and 24 depressed lesions. Table 1 lists the demographic and clinicopathologic characteristics according to macroscopic subtypes. There was no significant difference in age and gender distribution. A higher percentage of proximal location was noted for LSTs. The tumor size was relatively smaller for depressed lesions. Severe histological changes such as high-grade dysplasia, carcinoma in situ and invasive cancer were not noted for polypoid tumors.

**Table 1:** **Basic demographic characteristics of studied colorectal neoplasms**

	**Polypoid**	**LST-G**	**LST-NG**	**Depressed**
**Lesion no./patient no.**	**34/26**	**16/15**	**20/20**	**24/24**
Gender(M/F)	18/8	6/9	15/5	21/3
Mean age	61.0	67.7	64.9	61.3
Proximal/distal	16/18	13/3	16/4	12/12
Mean size ± SD (cm)	1.58	2.21	1.99	0.89
Histopathology				
HGD	0	2	4	3
CIS	0	2	3	0
Invasive cancer	0	2	3	3

HGD: high-grade dysplasia; CIS: carcinoma in situ.

### Mutational analyses in polypoid, LST and depressed neoplasms

**Figure 2:**
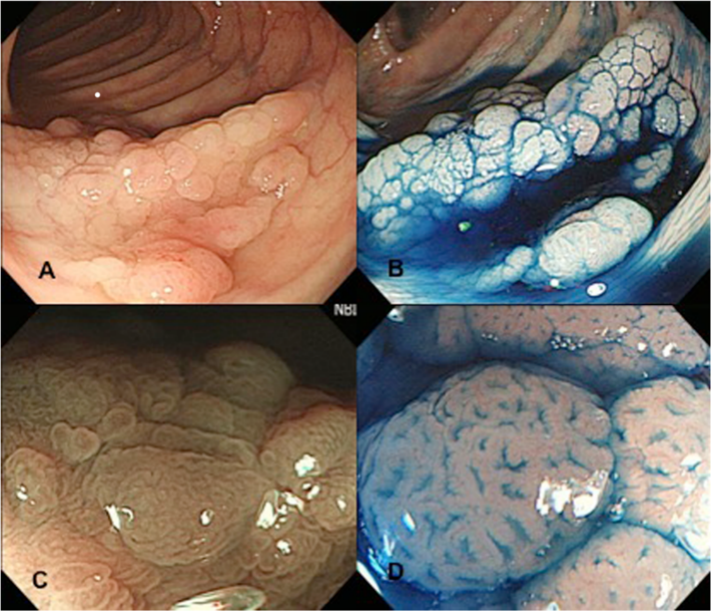
**A case of BRAF positive LST-G. A** and **B**: Conventional view and chromoendoscopy dye-spraying with indigo-carmine revealed 0-IIa (LST-G) lesion. **C**: NBI with magnifying observation revealed no obvious capillary mesh. **D**: Magnifying observation revealed type II pit pattern.

The mutational profile of all the specimens is summarized in Table 2. There was no mutation identified for EGFR, HER2 and AKT genes. KRAS mutation (codon 2) was noted more frequently in LST (13/36, 36.1%) than polypoid neoplasms (5/34, 14.7%, p = 0.041). When comparing LST-G with LST-NG, LST-G had a significantly higher frequency of KRAS mutation than in LST-NG. (9/16, 56.3% *vs.* 4/20, 20.0%, p = 0.024). BRAF mutation (V600E) was found in 2 of 36 (5.6%) LSTs and 1 of 34 (2.9%) polypoid lesions (Table 3). The two LST lesions with BRAF mutation were macroscopically classified as granular type and pathologically proven to be serrated adenoma (Figure 2). In contrast, the polypoid tumor with BRAF mutation was confirmed as traditional serrated adenoma. PIK3CA mutation (exon 9 E545K) was identified only in LST (5/36, 13.9%). Mutations in KRAS, BRAF or PIK3CA occurred in a mutually exclusive manner. All mutations were absent in the specimens obtained from depressed type neoplasms.

**Table 2:** **Mutation rates in different macroscopic subtypes of colorectal neoplasms**

**Mutation**	**LST(%)(n = 36)**	**Polypoid(%)(n = 34)**	**Depressed(%)(n = 24)**
KRAS	13/36 (36.1%)	5/34 (14.7%)	0/24 (0%)
BRAF	2/36 (5.6%)	1/24 (2.9%)	0/24 (0%)
PIK3CA	5/36 (13.9%)	0/34 (0%)	0/24 (0%)
AKT	0/36 (0%)	0/34 (0%)	0/24 (0%)
EGFR	0/36 (0%)	0/34 (0%)	0/24 (0%)
HER2	0/36 (0%)	0/34 (0%)	0/24 (0%)

**Table 3:** **Comparison of BRAF mutation in lesions with different macroscopic types**

**Morphology**	**BRAF (+)**	**BRAF (−)**	**p-value**
Polypoid (n = 34)	1*	33	1.00
LST (n = 36)	2*	34	
Depressed (n = 24)	0	24	-

*Pathologically turned out to have serrated change.

## Discussion

A wealth of data has revealed CRCs defined by distinct molecular and pathologic features correspond with differing responses to particular chemotherapy and clinical outcomes. The increasing recognition of distinct clinicopathologic behaviors between polypoid and non-polypoid colorectal polyps had added to the need for clarification of molecular pathogenesis of these different subtypes of neoplasm. The current study represents the first comprehensive and concurrent analysis of activation mutations in EGFR network. We found flat lesions, especially granular type LST, displayed a higher frequency of KRAS and PIK3CA mutations as compared to those of polypoid tumors. Furthermore, no mutations were detected in depressed tumors, indicating existence of different molecular pathways for these clinically more aggressive lesions. Our findings support the notion that different macroscopic colorectal polyps may have distinct pathogenesis.

KRAS is a GTPase protein that is activated by EGFR and other cell surface growth factor receptors and activation of EGFR/KRAS/BRAF pathway plays a key role in the carcinogenesis of several malignancies. Mutations in KRAS can be identified in 30-40% of colorectal cancers [13,14] and mutated KRAS is constitutively active independent of EGFR signaling. The presence of a KRAS mutation may predict lack of response to EGFR inhibitors in metastatic CRC [11,13,14]. In this study, we demonstrated that frequency of KRAS mutation was higher in LST-G than in LST-NG or polypid tumors. These findings were in agreement with the study of Sugimoto and colleagues [29]. In their study, they found LST-G displayed more frequent KRAS mutations and LST-NG had more nuclear accumulation of beta-catenin and expression of MYC. Similar observations of higher mutation rate of KRAS in LST-G have also been documented by Hiraoka and Mukawa et al. [30,31] The KRAS mutation in LST-G may vary according to different neoplasm location. Kaji et al. have reported that LST-G in the proximal colon was significantly associated with KRAS mutation [32]. Our study failed to analyze the mutation in distinct-morphology neoplasm with different location because of limited sample size, which is the limitation of our study. Collectively, subtypes of LST could have different molecular characteristics. Because LST in general could be detected in earlier stage and resected by endoscopy, whether these tumors, when become advanced, might have different responses to treatment remains to be further investigated.

BRAF is a downstream molecule of KRAS and mutation of BRAF V600E was detected in 5-10% of CRC [20,23]. Tumors with BRAF mutation are microsatellite instable, predominantly located in proximal colon, arising from serrated adenoma and have poor prognosis and unsatisfactory response to EGFR inhibitor [20,24,33]. In this study, we found two cases in LST and one case in polypoid tumors displayed BRAF mutations. Reevaluation of their pathology revealed they belonged to sessile serrated adenoma and traditional serrated adenoma respectively. Serrated pathway has recently been considered a separate one from traditional adenoma-adenocarcinoma pathway because of their characteristic flat appearance and distinct molecular alterations. These tumors were more frequently overlooked by endoscopists than traditional adenomas and regarded as an important cause of interval cancer [34-36]. The increasing complexity of macroscopic and histologic classification of colorectal polyps suggested communications and consensus between endoscopists and pathologists are crucial for further investigation of molecule profiles of CRCs.

The PIK3CA gene encodes a lipid kinase regulating signaling pathway downstream of the EGFR alongside with KRAS. Mutations of PIK3CA occurred in 15-20% of CRC and were associated with poor prognosis among curative resected CRCs [17,22]. The role of PIK3 mutations in determining EGFR inhibitor remains controversial. Two different reports showed discrepant results [18,21]. In contrast to CRC, the data of PIK3CA mutations in colorectal polyps remained few. Velho et al. have demonstrated one of 17 (5.6%) colorectal polyps had PIK3CA mutation [16]. However, no macroscopic classification was mentioned in their study. In our study, we found PIK3CA mutations occurred predominantly in LST. Our results reemphasized the importance of macroscopic subtyping in the investigation of genetic alterations of colorectal neoplasm.

Depressed colorectal lesions, in contrast to polypoid tumors, tended to develop high-grade dysplasia or submucosal invasive cancer when they were small. The aggressive behavior and characteristic morphology suggested that they may follow a different carcinogenic pathway to flat elevated or protruding adenomas. Compared to more and more endoscopic and clinicopathologic researches for depressed colorectal tumors, investigations of genetic alterations remain scanty [37,38]. Previous studies for these tumors showed no mutations in KRAS and high frequencies of p53 expression by immunohistochemistry [37]. Through PCR-based pyrosequencing, Konda et al. have reported that mutation in KRAS and BRAF occurred in 16% and 11% of depressed colorectal neoplasms respectively [39]. There was a discrepancy between this study and ours. In our study, we did not find any unique genetic changes regarding EGFR network, including KRAS and BRAF, in depressed colorectal neoplasms. This discrepancy may be attributed to low tumor content among our depressed colorectal neoplasms. The lower tumor content may lead to higher false negative result. Moreover, only 3 HGD and 3 T1 cancers occurred in our total 24 depressed colorectal neoplasms. In study conducted by Konda et al., all the 19 depressed lesions harbored HGD or invasive cancers. Therefore, the more advanced histology may contribute to more genetic alterations. Finally, the sample size in both studies was limited. Only 3 and 2 depressed colorectal neoplasms had mutations in KRAS and BRAF respectively. Further researches with larger sample size and in a multi-center setting are mandatory to elucidate the relevant genetic or epigenetic changes of this special subset of CRCs.

The investigation of genetic alterations in relation to different macroscopic subtypes may also provide new insights into CRC screening. Previous studies in this field have reported the usefulness of stool DNA testing in experimental settings but only modest or unsatisfactory sensitivity for cancer and advanced adenoma were observed in population-based study [40]. Superficial neoplasms, including fat and depressed lesions, are good candidates for the target of screening and endoscopic treatment because it remains non-invasive until fairly large size [7,41]. Development of molecular probe with combination of variable molecular marker is an attractive field which may enable detection of such a flat neoplasm much easier [42]. Several studies have used fluorescently labeled antibodies against epitopes that are commonly overexpressed in most GI cancers, such as vascular endothelial growth factor or epidermal growth factor receptor (EGFR) [43]. If such probe for superficial colorectal neoplasm is developed, it will be of great help for improving detectability during colonoscopy. To make this scenario fulfilled, elucidations of molecular pathogenesis in different subtypes of CRC are crucial.

In summary, our findings provide further insights into the genetic alterations of colorectal neoplasms with respect to distinct macroscopic morphology. The mutational profile of KRAS, and PIK3CA in LSTs is different from protruded lesions and different subtypes of LSTs display distinct mutations. LST-G with BRAF mutation is more likely to be a sessile serrated adenoma. No specific activating mutation in EGFR network is observed in the depressed lesions although these tumors behave more aggressive than protruded adenomas and LSTs. Further molecular studies of CRCs should take macroscopic subtypes into consideration and highlight the importance of consensus and communication between endoscopic and pathologic diagnosis.

## Conclusion

Three different macroscopic subtypes of colorectal neoplasms display distinct carcinogenetic pathways in EGFR networking. Further molecular studies of CRCs should take macroscopic subtypes into consideration and highlight the importance of consensus and communication between endoscopic and pathologic diagnosis.

## Abbreviations

CRC: Colorectal cancer
LST: Laterally spreading tumor
EGFR: Epidermal growth factor receptor
LST-G: Laterally spreading tumor, granular type
LST-NG: Laterally spreading tumor, non-granular type
HGD: High-grade dysplasia
CIS: Carcinoma in situ
PCR: Polymerase chain reaction
